# Predicting cerebral edema in patients with spontaneous intracerebral hemorrhage using machine learning

**DOI:** 10.3389/fneur.2024.1419608

**Published:** 2024-10-03

**Authors:** Jiangbao Xu, Cuijie Yuan, Guofeng Yu, Hao Li, Qiutong Dong, Dandan Mao, Chengpeng Zhan, Xinjiang Yan

**Affiliations:** ^1^The Quzhou Affiliated Hospital of Wenzhou Medical University, Quzhou People’s Hospital, Quzhou, China; ^2^Postgraduate Training Base Alliance of Wenzhou Medical University, Wenzhou, China; ^3^Wenzhou Institute, University of Chinese Academy of Sciences, Wenzhou, China

**Keywords:** SICH, cerebral edema, random forest, GDBT, XGBoost

## Abstract

**Background:**

The early prediction of cerebral edema changes in patients with spontaneous intracerebral hemorrhage (SICH) may facilitate earlier interventions and result in improved outcomes. This study aimed to develop and validate machine learning models to predict cerebral edema changes within 72 h, using readily available clinical parameters, and to identify relevant influencing factors.

**Methods:**

An observational study was conducted between April 2021 and October 2023 at the Quzhou Affiliated Hospital of Wenzhou Medical University. After preprocessing the data, the study population was randomly divided into training and internal validation cohorts in a 7:3 ratio (training: *N* = 150; validation: *N* = 65). The most relevant variables were selected using Support Vector Machine Recursive Feature Elimination (SVM-RFE) and Least Absolute Shrinkage and Selection Operator (LASSO) algorithms. The predictive performance of random forest (RF), GDBT, linear regression (LR), and XGBoost models was evaluated using the area under the receiver operating characteristic curve (AUROC), precision–recall curve (AUPRC), accuracy, F1-score, precision, recall, sensitivity, and specificity. Feature importance was calculated, and the SHapley Additive exPlanations (SHAP) and Local Interpretable Model-Agnostic Explanations (LIME) methods were employed to explain the top-performing model.

**Results:**

A total of 84 (39.1%) patients developed cerebral edema changes. In the validation cohort, GDBT outperformed LR and RF, achieving an AUC of 0.654 (95% CI: 0.611–0.699) compared to LR of 0.578 (95% CI, 0.535–0.623, DeLong: *p* = 0.197) and RF of 0.624 (95% CI, 0.588–0.687, DeLong: *p* = 0.236). XGBoost also demonstrated similar performance with an AUC of 0.660 (95% CI, 0.611–0.711, DeLong: *p* = 0.963). However, in the training set, GDBT still outperformed XGBoost, with an AUC of 0.603 ± 0.100 compared to XGBoost of 0.575 ± 0.096. SHAP analysis revealed that serum sodium, HDL, subarachnoid hemorrhage volume, sex, and left basal ganglia hemorrhage volume were the top five most important features for predicting cerebral edema changes in the GDBT model.

**Conclusion:**

The GDBT model demonstrated the best performance in predicting 72-h changes in cerebral edema. It has the potential to assist clinicians in identifying high-risk patients and guiding clinical decision-making.

## Introduction

Spontaneous intracerebral hemorrhage (SICH) is a prevalent subtype of stroke, with a mortality rate significantly higher than ischemic stroke. Approximately 20–30% of SICH patients die within 3 months (1–3). The high incidence and mortality rates pose a significant threat to public health (4, 5). Cerebral edema, a common complication of SICH, involves the accumulation of excess water in the brain tissues adjacent to the hemorrhage. This can lead to severe consequences, including compromised blood flow, intracranial pressure shifts, and neuronal damage (6, 7). Timely identification of edema development and its influencing factors is crucial for optimizing patient care, allocating resources effectively, and reducing healthcare costs. Cerebral edema typically appears within 24–72 h after bleeding, peaks 2–7 days later, and can persist for up to 2 weeks. To monitor edema progression, patients with SICH undergo head CT scans at admission, 24 and 72 h post-admission. Subsequent scans may be ordered based on clinical changes. This study aimed to develop and validate machine learning models capable of predicting changes in cerebral edema within the first 72 h following SICH (8).

Over the past few years, advances in imaging omics have refined the use of CT scans for evaluating brain edema (9). In addition, machine learning algorithms have demonstrated significant promise in predicting medical outcomes and complications, aiding clinicians in making informed decisions and enhancing patient care (10–13). These advancements inform the development of accurate and reliable prognostic models to identify patients at risk of severe cerebral edema, enabling healthcare providers to implement targeted preventive strategies and interventions.

This study sought to develop and validate a machine learning-based prognostic model that could evaluate the progression patterns and influencing factors of cerebral edema, considering various patient attributes and clinical determinants. We aimed to compare the predictive accuracy and clinical utility of different machine learning algorithms. Ultimately, our goal was to provide clinicians with valuable tools for the early identification of patients at risk of severe cerebral edema, enabling the implementation of targeted preventive strategies to reduce its prevalence.

## Methods

This study adhered to the Strengthening the Reporting of Observational Studies in Epidemiology (STROBE) reporting guidelines.

### Data source

This observational study was conducted between April 2021 and October 2023 at the Quzhou Affiliated Hospital of Wenzhou Medical University. The study protocol was approved by the hospital’s ethical board (reference number: LW2023-163) and adhered to the principles of the Declaration of Helsinki. All patients provided informed consent through their relatives, and no patient data were used in a way that could pose a risk to them. Patients with SICH were included if they met the following criteria: (1) admission within 72 h after first-ever stroke; (2) SICH confirmed by head computerized tomography (CT) scan; (3) hospitalization within 24 h after the onset of stroke symptom; and (4) age of 18 years or greater. Exclusion criteria encompassed the following:(1) secondary brain bleeding as a result of congenital or acquired coagulation abnormalities, hemorrhagic transformation of cerebral infarction, moyamoya disease, cerebral aneurysm, and arteriovenous malformation or tumor; (2) primary intraventricular bleeding; (3) presence of previous neurological diseases, such as brain tumors and severe head trauma; and (4) coexistence with severe systemic diseases, for example, malignancies, immune deficiency syndromes, and severe heart, liver, lung, or kidney dysfunction.

A total of 215 patients presented to the emergency department with suspected SICH, which was confirmed by head CT scans. All CT scans were conducted following the radiology department’s protocol by radiologists blinded to clinical information. To ensure data relevance, we collected 51 basic patient characteristics at admission (detailed in Supporting Material 1). In addition, we gathered 38 imaging characteristics, including clinical review times at 6, 24, 72 h, and subsequent hours until the absence of hematoma and edema was confirmed by two senior doctors (Supporting Material 1).

The cerebral edema volume was calculated using two methods: (1) Image-based analysis: The boundaries of hematoma and edema were delineated on CT scans using image browser measurement software. The hematoma volume was calculated by summing the areas of each layer, while the edema volume was determined by subtracting the hematoma volume from the combined volume of hematoma and surrounding brain edema. (2) Formula-based analysis (verification): The hematoma length and width in the maximum plane were measured as A and B, respectively, and the thickness (number of layers, C) was calculated. The hematoma volume was calculated as 1/2 ABC, with layers categorized as 75% (layer 1), 75–25% (1/2 layer), and < 25% (excluded). The edema volume was determined as the difference between the combined volume of blood and edema and the hematoma volume. Absolute hematoma and edema volumes were assessed at each time point. The primary outcome measure was defined as an increase in cerebral edema volume between baseline and repeat imaging of more than 6 mL or a relative increase of >33% within 72 h (14–17). To optimize statistical power and minimize bias, multiple imputation using random forests was employed to supplement missing values. The imputed data were then randomly stratified into training (*N* = 150) and validation cohorts (*N* = 65) in a 7:3 ratio.

### Feature selection

To prevent variable misselection, we employed a rigorous variable selection approach using a training cohort to identify the most relevant predictors for constructing a predictive model. Initially, pairwise Pearson’s correlation matrices were used to assess the collinearity of clinical variables. Collinearity occurs when two or more predictors exhibit a strong correlation (*r* > 0.8), complicating the evaluation of each variable’s unique contribution to the outcome. Therefore, we have chosen to remove the more readily available variables from the collinear variables. Subsequently, we used the Minimum Absolute Shrinkage and Selection Operator (LASSO) and the SVM-RFE algorithm in a two-step process. LASSO is a regularization technique that performs variable selection and coefficient estimation by applying constraints to the sum of the absolute values of the model parameters. This process causes some of the coefficients to be narrowed down to zero, effectively excluding them from the final model. Then, the SVM-RFE algorithm was used for further variable selection. The SVM-RFE algorithm enables the machine learning algorithm to continuously reduce the number of features, verify the performance of the model, and finally achieve the optimal number of features for screening. By using the SVM-RFE algorithm, we obtain another important set of predictors. Finally, the intersection of predictors determined by the LASSO and SVM-RFE algorithms is employed to ensure that only the most relevant and robust variables are included in the development of our predictive models. This combined approach aims to improve the accuracy and generalizability of the model while reducing the risk of overfitting or including irrelevant predictors.

### Model development and validation

We employed four machine learning classifiers—extreme gradient boosting (XGBoost), random forest (RF), linear regression (LR), and gradient-boosted decision trees (GDBT) (18–21)—to develop predictive models for the risk of 72-h brain edema growth. All models incorporated the same input variables. Grid and random hyperparameter searches were conducted on the training data to identify optimal hyperparameters for each model, with performance evaluated using the area under the receiver operating characteristic curve (AUROC), precision–recall curve (AUPRC), F1 score, precision, recall, sensitivity, and specificity. To interpret the best-performing model, Shapley Additive exPlanations (SHAP) (22) and Local Interpretable Model-Agnostic Explanations (LIME) (23) were applied to provide consistent and locally accurate variable importance values, enhancing our understanding of the model’s predictive capabilities.

### Dataset selection

**Figure 1:**
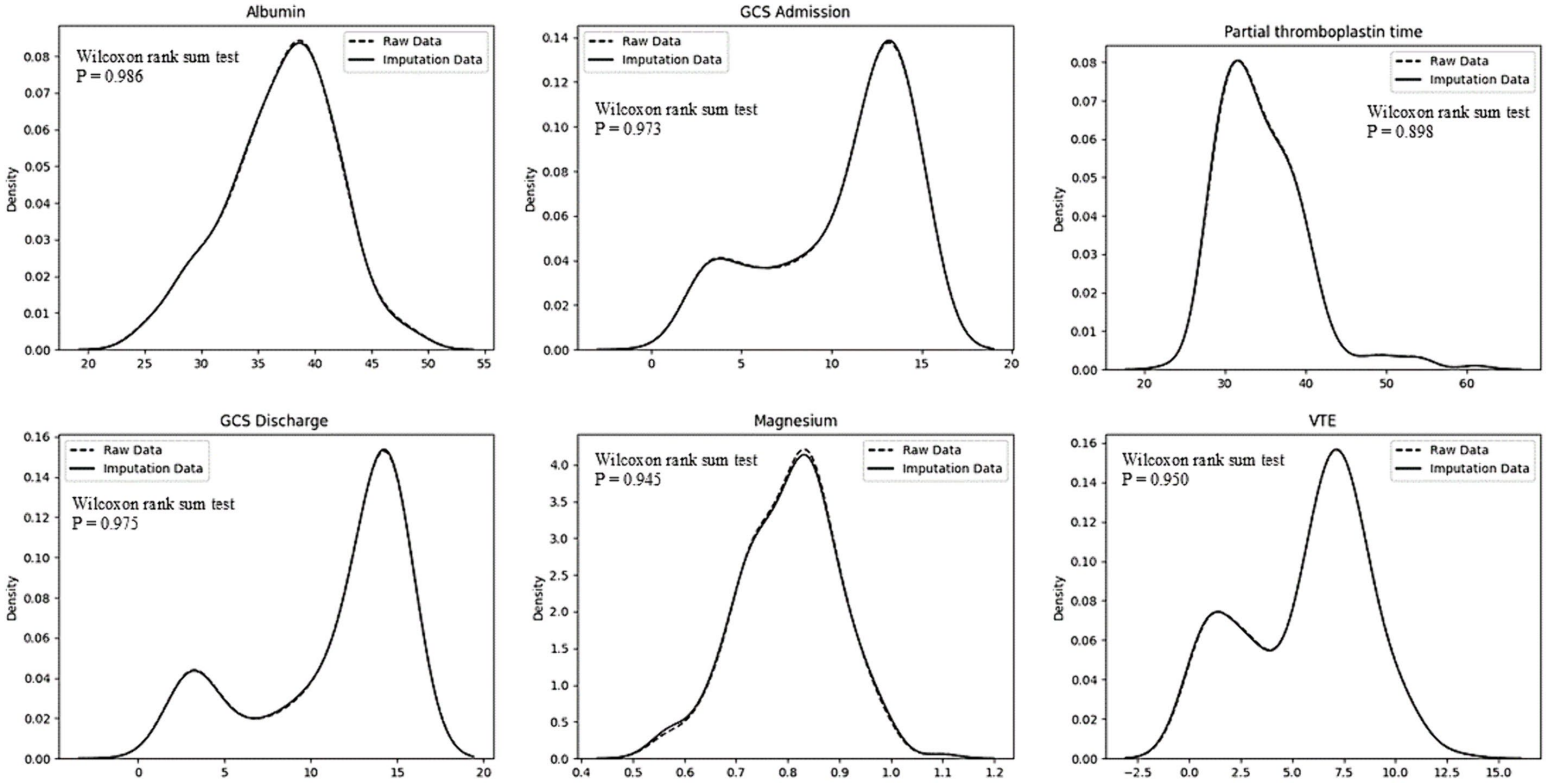
Dataset selection plots.

Given the limited sample size in this study, which can introduce bias, a 5-fold cross-validation was employed to ensure objectivity and minimize sampling bias. To select the dataset with the greatest statistical significance, a Wilcoxon rank-sum test was performed. The dataset with the largest *p*-value was chosen as illustrated in Figure 1.

### Tuning of hyperparameters

#### XGBoost

**Figure 2:**
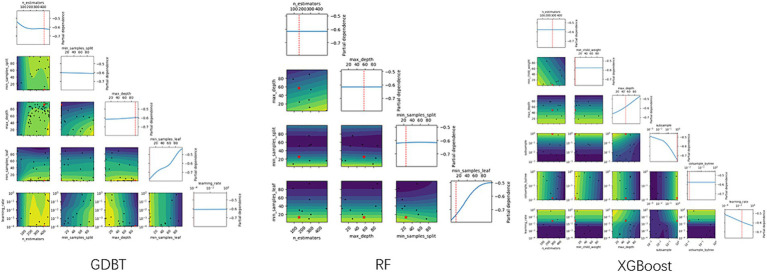
Hyperparameter selection plots.

XGBoost, a widely used and powerful ensemble technique, is based on the gradient boosting framework. It combines the predictions of multiple weak learners, primarily decision trees, to create a more accurate and robust model. XGBoost implements machine learning algorithms within the Gradient Boosting framework. The optimal parameters were determined using the “xgboost” package and 5-fold cross-validation, as illustrated in Figure 2.

#### Random forest

Random forest algorithms utilize tree-based models, combining multiple decision trees through bootstrapping to improve predictive accuracy (19). The optimal number of trees was determined using 5-fold cross-validation with the “randomForest” package, as illustrated in Figure 2.

#### Linear regression

Linear regression is a widely used statistical method for modeling binary outcomes. The most common approach is least squares, which aims to minimize the average squared error between predicted and observed values. To select optimal variables and construct an LR model, we employed backward stepwise regression based on the Akaike information criterion. The “MASS” package in R software was utilized to fit the model (24).

### Gradient-boosted decision tree (GBDT)

The GBDT model iteratively calculates residuals at each step and establishes the model by moving in the negative gradient direction of these residuals. GBDT’s powerful, flexible, efficient, and accurate predictive capabilities have made it a popular machine-learning algorithm for analyzing and processing abstract data. Optimal parameters for the GBDT model were determined using the “GBDT” package and 5-fold cross-validation, as illustrated in Figure 2.

### Statistical analysis

Prior to formal analysis, the Kolmogorov–Smirnov test was used to assess data distribution. Continuous variables were analyzed using either the independent *t*-test (for normally distributed data) or the Mann–Whitney *U*-test (for non-normally distributed data) and were presented as mean ± standard deviation (SD) or median with interquartile range (IQR), respectively. Categorical variables were analyzed using the chi-square test for large samples or Fisher’s exact test for small samples and are expressed as frequencies (percentages). To compare the area under the curve (AUC) of the different models statistically, the DeLong test was used. All statistical tests were two-tailed, and a *p* < 0.05 was considered statistically significant. In addition, the study adhered to the rule of thumb of having at least 10 events per variable for robust analysis. Statistical analyses were performed using R (version 4.2.2; R Foundation for Statistical Computing) and Python (version 3.9.0; Python Software Foundation).

## Results

### Patient characteristics

The dataset comprised information on 215 patients with SICH, including 949 imaging CT scans. The total number of CT scans was determined at specific time frames: 6, 24, 72 h, and subsequent hours until the absence of hematoma and edema was confirmed by two senior doctors. Of these patients, 86 (40%) exhibited cerebral edema expansion (edema volume increased by more than 6 mL or by >33% relative to the last measurement) within 72 h. The cohort included 143 male (66.5%) and 72 female (33.5%) patients, with 60 male (69.8%) and 26 female (30.2%) patients experiencing dilated cerebral edema. No significant differences were observed in baseline characteristics between the training and validation groups. Tables 1, 2 provide detailed baseline patient characteristics.

**Table 1:** Summary table of categorized data.

Characteristics	Totals (*n* = 215)	Training cohort (*n* = 150)	Validation cohort (*n* = 65)	*P*-value
Sex	143 (0.665)	101 (0.673)	42 (0.646)	0.818
History of stroke	58 (0.270)	40 (0.267)	18 (0.277)	1.000
History of diabetes	29 (0135)	18 (0.120)	11 (0.169)	0.451
History of atrial fibrillation	12 (0.056)	8 (0.053)	4 (0.062)	1.000
History of coronary heart disease	92 (0.428)	71 (0.473)	21 (0.323)	0.058
History of hypertension	183 (0.851)	124 (0.827)	59 (0.908)	0.185
History of smoking	69 (0.321)	53 (0.353)	16 (0.246)	0.165
History of alcohol consumption	73 (0.340)	53 (0.353)	20 (0.308)	0.623
History of hyperlipidemia	4 (0.019)	1 (0.007)	3 (0.046)	0.156
Cranial decompression Hematoma removal	67 (0.312)	43 (0.287)	24 (0.369)	0.298
Ventricular drainage	71 (0.330)	44 (0.293)	27 (0.415)	0.112
Hemostatic therapy	200 (0.930)	141 (0.940)	59 (0.908)	0.574
Cranial pressure-lowering therapy	194 (0.902)	138 (0.920)	56 (0.862)	0.282
Antihypertensive treatment	208 (0.967)	146 (0.973)	62 (0.954)	0.748
Antiemetic and antacid	211 (0.981)	148 (0.987)	63 (0.969)	0.749
Lipid-lowering therapy	20 (0.093)	17 (0.113)	3 (0.046)	0.193

**Table 2:** Summary table of continuous variable data.

Characteristics	Totals (*n* = 215)	Training cohort (*n* = 150)	Validation cohort (*n* = 65)	*P*-value
Age	66.0 (56.0, 75.0)	66.5 (56.0, 76.75)	66.0 (56.0, 73.0)	0.531
Time from onset to first imaging	6.0 (4.0, 10.5)	5.5 (4.0, 10.0)	6.0 (4.0, 12.0)	0.637
Systolic blood pressure	158.0 (142.0, 174.0)	158.5 (142.25, 174.0)	158.0 (142.0, 173.0)	0.723
Diastolic blood pressure	88.0 (78.0, 97.0)	87.0 (77.25, 97.0)	88.0 (78.0, 100.0)	0.647
VTE	6.0 (3.0, 8.0)	7.0 (3.0, 8.0)	6.0 (3.0, 7.0)	0.351
Hemoglobin	133.0 (122.0, 143.0)	133.0 (123.0, 144.0)	133.0 (121.0, 141.0)	0.436
Blood platelet count	176.0 (128.0, 212.5)	177.5 (135.5, 210.75)	169.0 (117.0, 218.0)	0.563
Partial thromboplastin time	33.4 (30.65, 37.3)	33.85 (30.725, 38.15)	32.7 (30.4, 36.0)	0.057
Prothrombin time (blood clotting enzyme)	17.1 (16.3, 17.7)	17.1 (16.3, 17.6)	17.1 (16.4, 17.8)	0.604
Prothrombin time	13.2 (12.6, 13.85)	13.2 (12.6, 13.85)	13.2 (12.8, 13.8)	0.845
Triglyceride	0.97 (0.705, 1.45)	0.95 (0.712, 1.438)	1.05 (0.69, 1.49)	0.567
Cholesterol	4.03 (3.42, 4.72)	3.925 (3.43, 4.742)	4.24 (3.41, 4.63)	0.460
High-density lipoprotein (HDL) cholesterol	1.22 (0.99, 1.45)	1.2 (0.982, 1.402)	1.26 (1.03, 1.5)	0.280
Glutamic-pyruvic transaminase	18.4 (13.0, 28.55)	18.9 (13.125, 28.575)	17.6 (12.6, 28.3)	0.963
Creatine kinase	106.3 (69.15, 184.05)	107.5 (69.6, 181.825)	103.3 (64.7, 188.7)	0.571
Lactate dehydrogenase	212.2 (184.75, 255.25)	209.2 (186.175, 250.85)	220.7 (183.8, 277.6)	0.271
Creatine kinase isoenzyme	20.8 (15.65, 27.55)	20.95 (16.2, 26.875)	20.1 (15.1, 29.5)	0.652
Magnesium	0.81 (0.74, 0.86)	0.805 (0.732, 0.86)	0.82 (0.75, 0.88)	0.232
Sugar	6.39 (5.31, 8.39)	6.375 (5.262, 8.278)	6.94 (5.41, 8.6)	0.356
Calcium	2.19 (2.1, 2.26)	2.19 (2.1, 2.278)	2.18 (2.1, 2.24)	0.629
Sodium	141.0 (139.15, 143.2)	141.1 (138.825, 143.1)	141.0 (139.9, 143.3)	0.310
Albumin	37.7 (34.25, 40.3)	37.95 (34.3, 40.6)	37.0 (34.1, 39.8)	0.387
C-reactive protein	4.26 (1.73, 17.005)	4.175 (1.762, 13.252)	4.35 (1.46, 19.2)	0.737
ADL admission	10.0 (0.0, 35.0)	10.0 (0.0, 35.0)	10.0 (0.0, 35.0)	0.808
ADL discharge	40.0 (0.0, 60.0)	37.5(0.0, 60.0)	50.0(0.0, 70.0)	0.172
Admission mRS score	4.0 (3.0, 5.0)	4.0 (4.0, 5.0)	4.0 (3.0, 5.0)	0.174
Mean_median_offset	0.0 (−0.517, 1.224)	0.0 (−0.375, 1.192)	0.0 (−0.533, 1.214)	0.957
Mean_cerebral subarachnoid hemorrhage volume	0.014 (0.0, 0.194)	0.018 (0.0, 0.167)	0.0 (0.0, 0.267)	0.563
Mean_subdural hemorrhage.3. Epidural hemorrhage4	0.0 (0.0, 0.0)	0.0 (0.0, 0.0)	0.0 (0.0, 0.125)	0.250
Mean_subdural hemorrhage	0.0 (0.0, 0.0)	0.0 (0.0, 0.0)	0.0 (0.0, 0.0)	0.809
Mean_CT5	0.0 (0.0, 0.0)	0.0 (0.0, 0.0)	0.0 (0.0, 0.0)	0.760
Mean_brainstem	0.0 (0.0, 0.4)	0.0 (0.0, 0.333)	0.0 (0.0, 0.5)	0.777
Mean_left_cerebellum	0.0 (0.0, 0.167)	0.0 (0.0, 0.125)	0.0 (0.0, 0.25)	0.413
Mean_left_basal_ganglia	0.25 (0.0, 1.0)	0.225 (0.0, 1.0)	0.25 (0.0, 1.0)	0.793
Mean_left_frontal_lobe	0.0 (0.0, 0.354)	0.0 (0.0, 0.333)	0.0 (0.0, 0.375)	0.861
Mean_left_temporal_lobe	0.2 (0.0, 0.854)	0.167 (0.0, 0.75)	0.25 (0.0, 1.0)	0.463
Mean_left_thalamus	0.0 (0.0, 0.388)	0.0 (0.0, 0.333)	0.0 (0.0, 0.5)	0.677
Mean_left_parietal	0.0(0.0, 0.6)	0.125(0.0, 0.6)	0.0(0.0, 0.75)	0.540
Mean_left_occipital_lobe	0.0 (0.0, 0.062)	0.0 (0.0, 0.0)	0.0 (0.0, 0.143)	0.245
Mean_right_cerebellum	0.0 (0.0, 0.167)	0.0 (0.0, 0.2)	0.0 (0.0, 0.125)	0.701
Mean_right_basal_ganglia	0.2 (0.0, 1.0)	0.25 (0.0, 1.0)	0.0 (0.0, 1.0)	0.527
Mean_right_frontal_lobe	0.0 (0.0, 0.333)	0.0 (0.0, 0.333)	0.0 (0.0, 0.25)	0.843
Mean_right_temporal_lobe	0.0 (0.0, 0.667)	0.0 (0.0, 0.667)	0.0 (0.0, 0.5)	0.321
Mean_right_thalamus	0.0 (0.0, 0.5)	0.0 (0.0, 0.575)	0.0 (0.0, 0.333)	0.584
Mean_right_parietal	0.0 (0.0, 0.5)	0.0 (0.0, 0.5)	0.0 (0.0, 0.25)	0.208
Mean_right_occipital_lobe	0.0 (0.0, 0.0)	0.0 (0.0, 0.0)	0.0 (0.0, 0.0)	0.514
Mean_fisher	0.5 (0.0, 1.31)	0.586 (0.0, 1.333)	0.429 (0.0, 1.167)	0.463

### Feature selection

**Figure 3:**
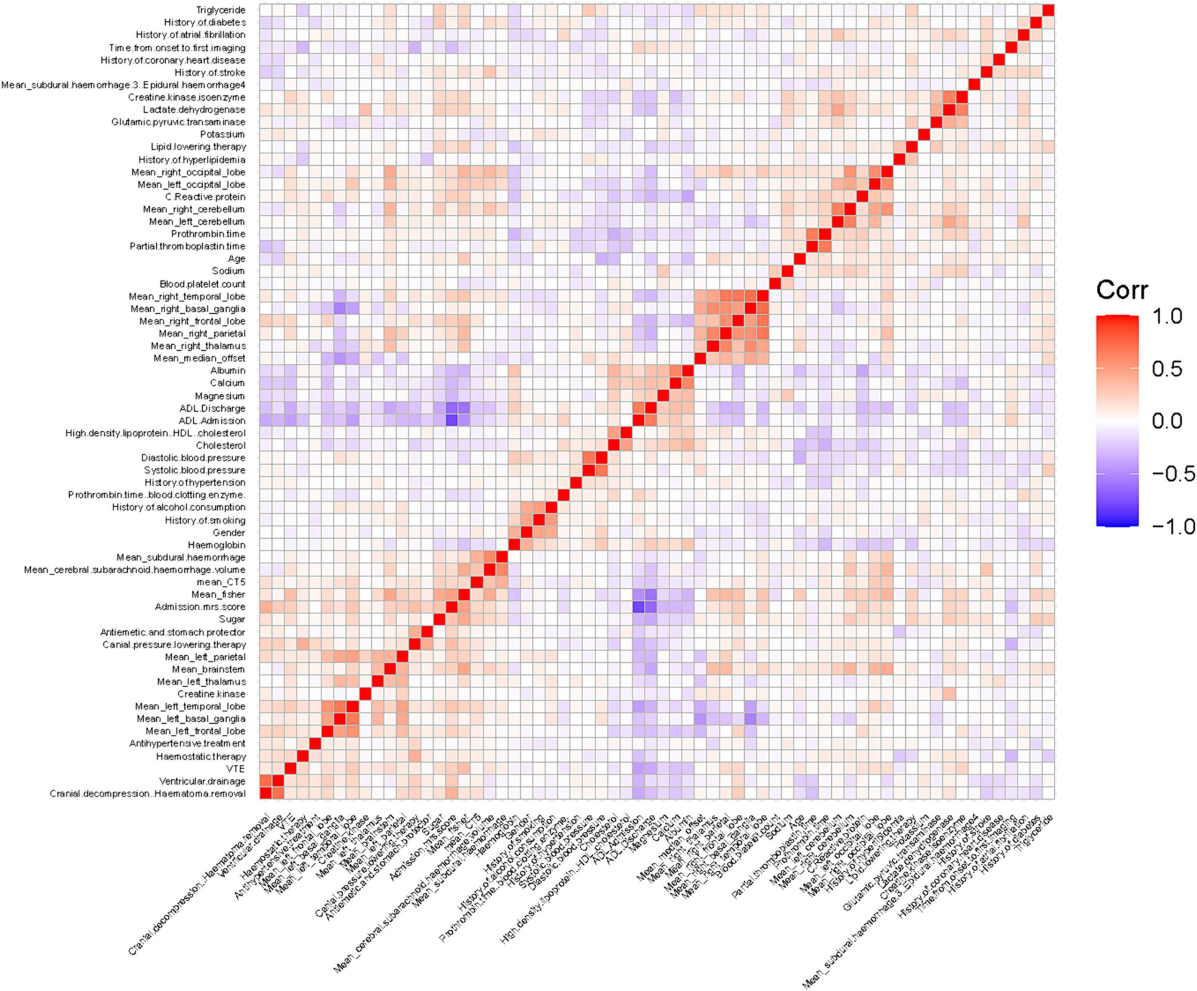
Pearson’s correlation matrix thermodynamic chart.

**Figure 4:**
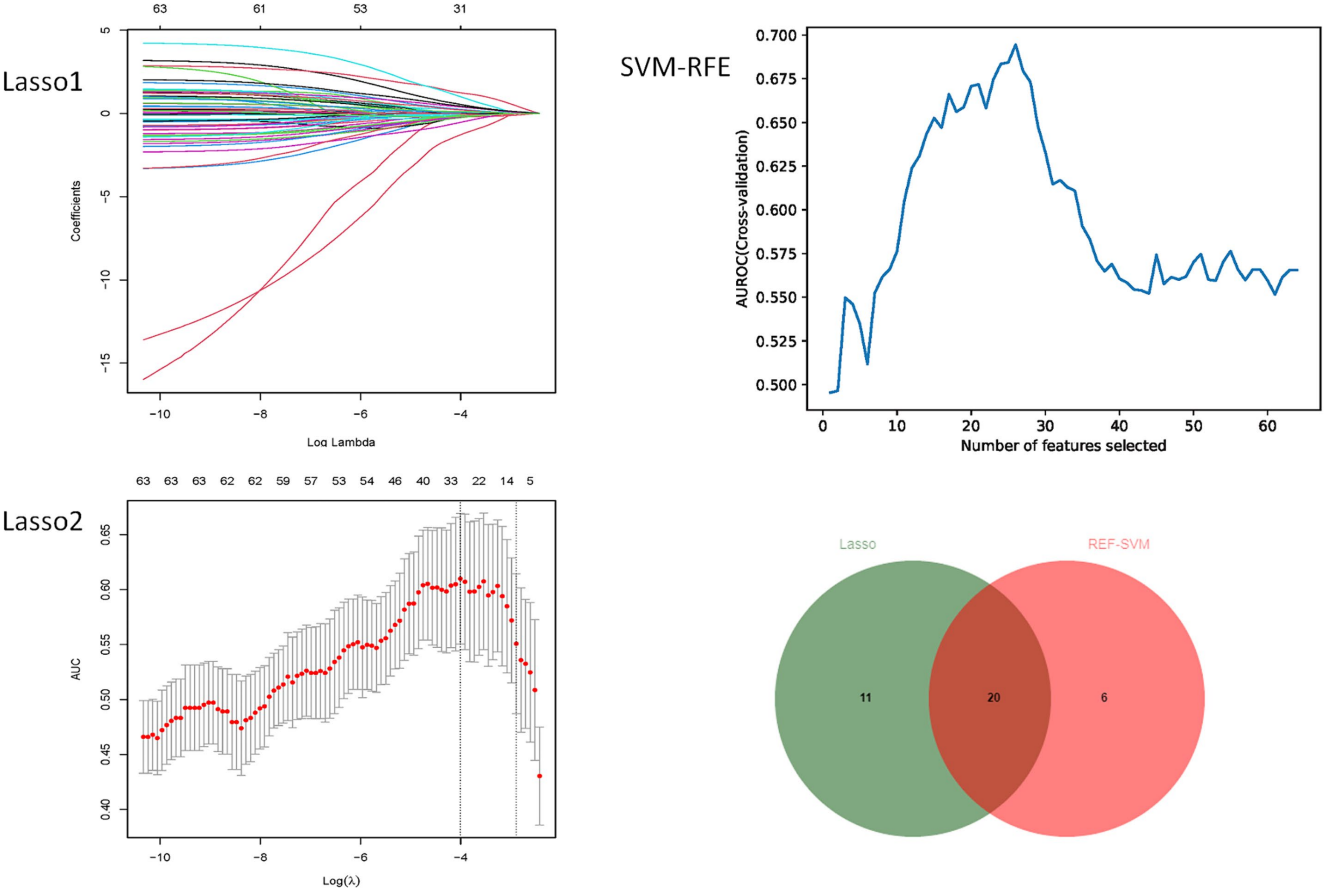
Variable screening diagram.

As shown in Figure 3, no pairwise Pearson’s correlations between continuous variables exceeded 0.8, indicating the absence of collinearity. Consequently, all variables were included in the subsequent feature selection process. SVM-RFE identified 31 important predictors (Supporting Material 1), while the LASSO regression algorithm selected 26 (Supporting Material 1). Ultimately, 20 factors emerged as significant predictors of the outcome (Figure 4), including sex, diabetes history, hypertension history, alcohol history, ventricular drainage, hemostatic treatment, decompressive craniectomy, antihypertensive treatment, antiemesis and antacid, HDL, cholesterol, alanine aminotransferase, serum magnesium, serum sodium, CRP, admission Barthel ADL Index, cerebral subarachnoid hemorrhage volume, subdural hemorrhage volume, and hemorrhage in the left cerebellum, left basal ganglia, or left parietal lobe. These selected features were integrated into four machine learning classifiers—GDBT, LR, RF, and XGBoost—to develop the predictive model.

### Tuning of hyperparameters

As shown in Table 3 and Figure 2, the optimal hyperparameters for the GDBT models were as follows: n_estimators (10–250), min_samples_split (2–25), max_features (0.1–0.999), max_depth (3–15), min_samples_leaf (1–25), and learning_rate (0.001–0.3). For the extreme gradient boosting (XGBoost) models, the optimal hyperparameters were as follows: n_estimators (10–250), min_samples_split (1–25), max_depth (3–15), subsample (0.001–1), colsample_bytree (0.01–1), and learning_rate (0.001–0.3). Finally, the optimal hyperparameters for the RF models were as follows: n_estimators (10–250), max_depth (3–15), min_samples_split (2–25), min_samples_leaf (1–25), and max_features (0.1–0.999).

**Table 3:** Summary table of the model parameters.

GDBT		XGBoost		RF	
n_estimators	(10, 250)	n_estimators	(10, 250)	n_estimators	(10, 250)
min_samples_split	(2, 25)	min_child_weight	(1, 25)	max_depth	(3, 15)
max_features	(0.1, 0.999)	max_depth	(3, 15)	min_samples_split	(2, 25)
max_depth	(3, 15)	subsample	(0.001, 1)	min_samples_leaf	(1, 25)
min_samples_leaf	(1, 25)	colsample_bytree	(0.01, 1)	max_features	(0.1, 0.999)
learning_rate	(0.001,0.3)	learning_rate	(0.001,0.3)		

### Development and validation of prediction models

**Figure 5:**
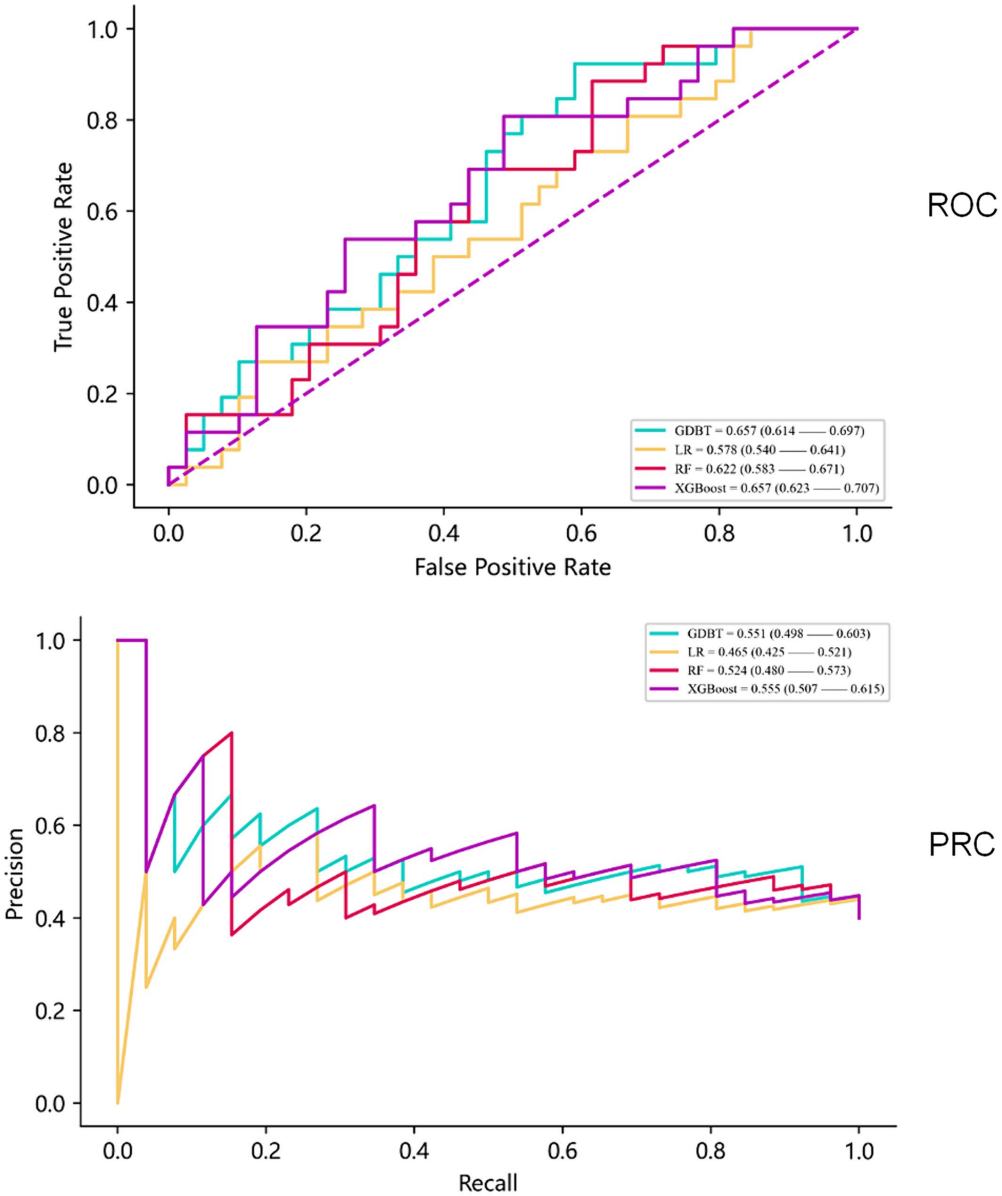
Receiver operating curves and precision–recall curves of the models.

When evaluating model performance on the validation cohort, our results demonstrated that the GDBT model, with an AUC value of 0.654 (95% CI: 0.611–0.699), outperformed the LR and RF models, which yielded AUC values of 0.578 (95% CI: 0.535–0.623, DeLong: *p* = 0.197) and 0.624 (95% CI: 0.588–0.687, DeLong: *p* = 0.236), respectively. Similarly, GDBT outperformed XGBoost, with an AUC of 0.660 (95% CI: 0.611–0.711, DeLong: *p* = 0.963). However, in the training set, GDBT (AUC = 0.603 ± 0.100) outperformed XGBoost (AUC = 0.575 ± 0.096). To mitigate the effects of random sampling, we repeated this process 50 times. Over-validation revealed that the LR model exhibited overfitting, performing poorly on the independent dataset despite good performance on the training set. In contrast, the GDBT model demonstrated greater stability and superior performance in both the training and validation sets compared to XGBoost. Based on these results, we selected the GDBT model for subsequent experiments, as summarized in Table 4. Receiver operating curves and precision–recall curves for the models are depicted in Figure 5.

**Table 4:** Model performance evaluation using training and validation cohorts.

Cohort	Model	AUROC	AUPRC	F1	Sensitivity	Specificity	Accuracy
Training (Mean ± SD)	GDBT	0.603 ± 0.100	0.501 ± 0.112	0.343 ± 0.146	0.839 ± 0.081	0.463 ± 0.187	0.663 ± 0.066
	LR	0.743 ± 0.094	0.630 ± 0.124	0.584 ± 0.106	0.737 ± 0.096	0.546 ± 0.112	0.708 ± 0.076
	RF	0.566 ± 0.100	0.450 ± 0.098	0.354 ± 0.126	0.733 ± 0.100	0.382 ± 0.128	0.609 ± 0.073
	XGBoost	0.575 ± 0.096	0.444 ± 0.091	0.323 ± 0.128	0.779 ± 0.087	0.388 ± 0.150	0.623 ± 0.069
Validation [median (95% CI)]	GDBT	0.654 (0.611, 0.699)	0.548 (0.503, 0.592)	0.444 (0.389, 0.502)	0.769 (0.733, 0.807)	0.526 (0.474, 0.578)	0.615 (0.584, 0.645)
	LR	0.578 (0.535, 0.623)	0.466 (0.423, 0.506)	0.424 (0.374, 0.477)	0.617 (0.573, 0.660)	0.424 (0.376, 0.465)	0.540 (0.497, 0.573)
	RF	0.624 (0.588, 0.687)	0.526 (0.485 ，0.584)	0.413 (0.365, 0.464)	0.667 (0.625, 0.735)	0.438 (0.400, 0.508)	0.556 (0.529, 0.605)
	XGBoost	0.660 (0.611, 0.711)	0.558 (0.501, 0.643)	0.450 (0.391, 0.515)	0.770 (0.720, 0.830)	0.531 (0.456, 0.630)	0.618 (0.575, 0.672)

### Model explainability

**Figure 6:**
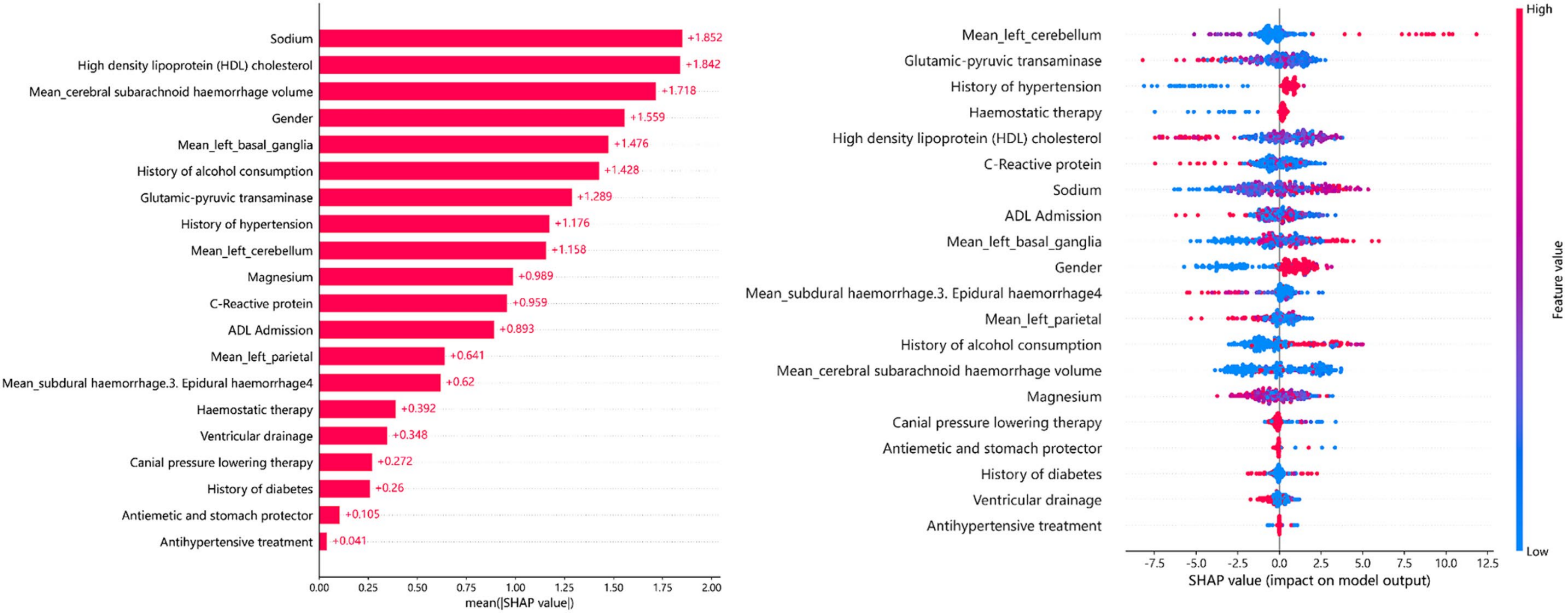
SHAP summary plot.

The SHAP summary plot (Figure 6) illustrates the relative importance of the 20 predictors in the GDBT model. We discovered that serum sodium, HDL cholesterol, subarachnoid hemorrhage volume, sex, and left basal ganglia hemorrhage volume were the five most significant features for predicting cerebral edema changes in the SHAP (GDBT) model.

**Figure 7:**
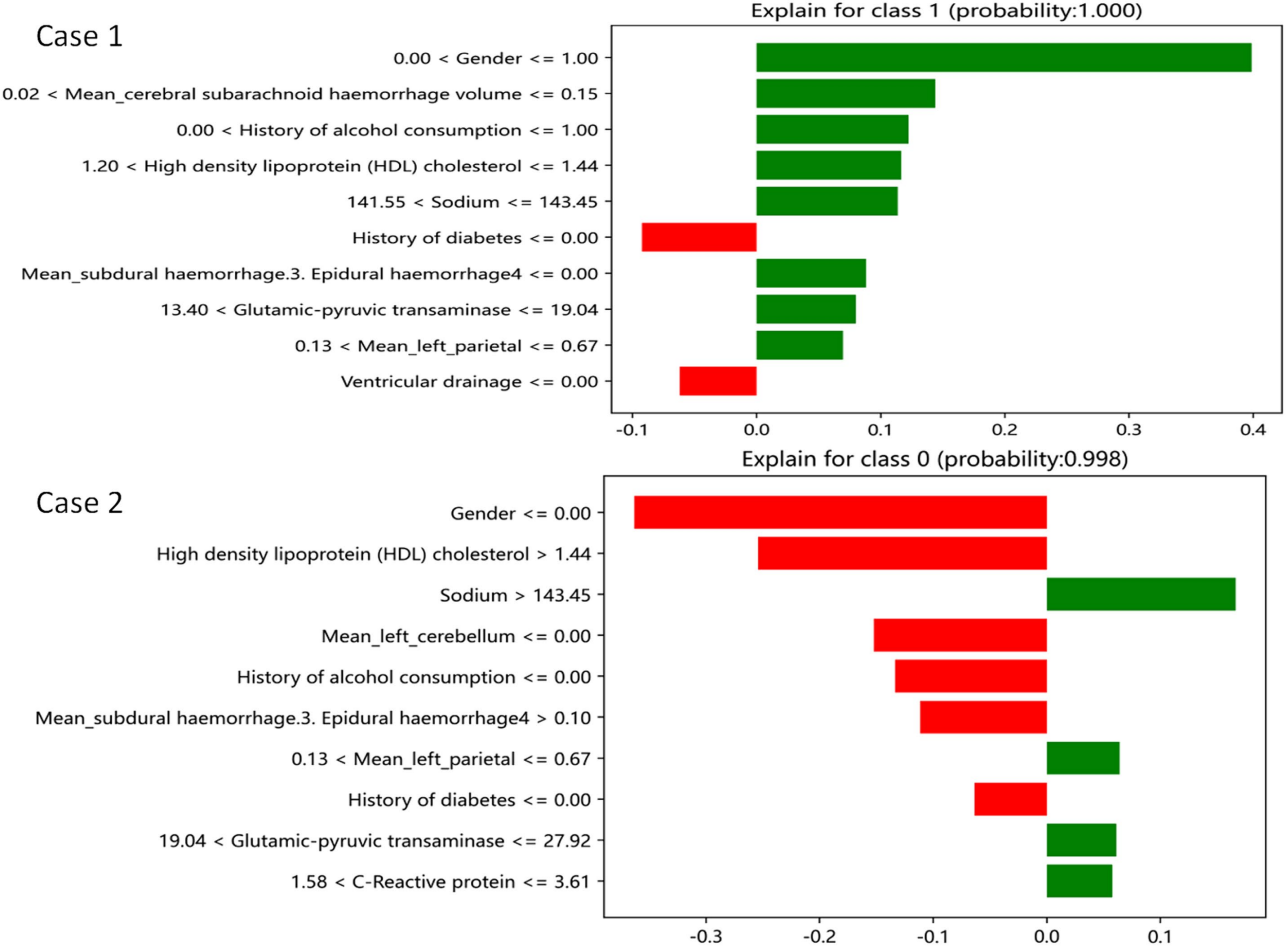
GDBT is explained by a locally interpretable model. Features with green bars favor the results, while those with red bars contradict the results. The X axle shows how much of each feature is added or subtracted from the patient’s final probability value (i.e., a feature with a weight of 0.3 is equivalent to a 30% change in the probability of the outcome). Class 1 represents increased edema, and class 0 represents no increased edema.

The LIME interpreter was applied to data generated by the GDBT model to examine classification outcomes. Each case’s feature weights are depicted in Figure 7, with green indicating factors favoring the outcome and red representing those opposing it. In case 1, the 100% predicted increase in edema was likely attributed to sex (male), cerebral subarachnoid hemorrhage volume within the range of 0.02–0.15 mL, alcohol use history, HDL levels between 1.2 and 1.44 mmol/L, serum sodium between 141.55 and 143.45 mmol/L, absence of subdural hemorrhage, alanine aminotransferase levels within 13.4–19.4 U/L, and left parietal lobe volume between 0.13 mL and 0.67 mL (favoring variables). However, no drinking history and no cerebral ventricular drainage were the opposite variables. Conversely, in case 2, the 99.8% prediction of no edema increase was likely due to serum sodium levels exceeding 143.45 mmol/L, left parietal bleeding volume within the 0.13–0.67 mL range, alanine aminotransferase levels between 19.04 U/L and 27.92 U/L, and CRP levels within 1.58–3.61 mg/L (favoring variables). Sex (male), HDL levels exceeding 1.44 mmol/L, and a subdural hemorrhage volume >0.1 mL were the opposite variables.

## Discussion

Hemorrhagic stroke, also known as cerebral hemorrhage, occurs when non-traumatic blood vessels in the brain rupture, leading to blood accumulation in the brain parenchyma. This condition constitutes 10–15% of all stroke cases and is characterized by rapid progression, severe neurological dysfunction, and a high mortality rate, particularly mortality rate during the acute phase (up to 100%). Increased intracranial pressure and cerebral herniation due to cerebral edema are major causes of death. Patients may also experience long-term neurological deficits, impacting their self-care ability and imposing substantial economic burdens on society and families. Early diagnosis and timely treatment are crucial for reducing mortality rates. To predict 72-h brain edema growth, we developed and validated machine-learning models using four different algorithms (GDBT, LR, RF, and XGBoost). Twenty key predictors were identified, and internal and external validation demonstrated the superior performance and clinical applicability of the GDBT model (25–28).

Our importance analysis identified serum sodium levels as the most significant predictor of 72-h brain edema growth risk, aligning with previous research (29–33). These findings support the role of serum sodium as a valuable prognostic indicator in brain edema. Previous studies suggest that edema around hematoma is predominantly vasogenic in the early stages, transitioning to cytotoxic edema later. Distal and contralateral edema is attributed to osmotic effects caused by the diffusion of edematous fluid and the accumulation of permeable substances within the bleeding area. Vasogenic cerebral edema results from blood–brain barrier impairment and increased permeability, leading to the leakage of plasma components, including sodium (Na^+^) and potassium (K^+^) ions. Cytotoxic edema arises from cytotoxic substances disrupting cell energy metabolism, leading to abnormal extracellular ion concentration gradients. Increased extracellular potassium ions are primarily removed through the blood–brain barrier via Na^+^-K^+^-ATPase-mediated Na^+^-K^+^ exchange, resulting in a net increase of cations. Our study demonstrated a correlation between lower serum sodium levels and increased edema volume. Potential explanations include the cytotoxic edema perspective: Despite constant serum sodium levels, cytotoxic substances may increase Na^+^ in edema fluid while decreasing it in plasma. Lower serum sodium levels may indicate more potent cytotoxic substances, leading to higher edema fluid osmotic pressure and increased edema volume.

Our importance analysis further identified high HDL values, hypertension history, alcohol history, and sex (male) as additional predictors of 72-h brain edema growth risk. Several studies and statistical analyses (34, 35) have reported that patients with cerebral hemorrhage accompanied by poorly controlled hypertension, alcohol consumption, or hyperlipidemia have a significantly higher likelihood of developing severe cerebral edema than healthy individuals. Histological studies have revealed that long-term hypertension can damage small-vessel wall structures. In addition, alcohol consumption, hyperlipidemia, and sex (male) are factors that can exacerbate this damage, contributing to morphological changes associated with cerebral hemorrhage and edema. Therefore, individuals with a history of hyperlipidemia, hypertension, alcohol consumption, or those who are male should be closely monitored for edema growth and receive timely treatment.

Our importance analysis further revealed that the volume of cerebral hematoma was the third most important factor associated with increased edema. In addition, cerebral ventricular drainage, hemostatic treatment, decompressive craniectomy, and antihypertensive treatment effectively reduced edema growth. The hematoma volume is a well-established marker influencing edema volume (36). Although it ranked third in our analysis, we speculate that this might be due to surgical interventions affecting hematoma volume, potentially altering the correlation between hematoma volume and edema growth. This could lead to a less consistent relationship between the two, making hematoma volume less consistently predictive of edema growth. However, further research is needed to confirm this hypothesis. Moreover, the demonstrated effectiveness of ventricle drainage, hemostasis, cranial pressure reduction, and antihypertensive treatment validates the reliability of our predictive model.

Our importance analysis identified CRP and ALT as factors promoting edema growth. Intracerebral hemorrhage is a common clinical condition characterized by rapid onset and progression, posing a significant threat to patient survival. Even if patients survive, they may experience adverse effects on multiple organ functions, leading to multiple organ failure syndrome and death. The primary cause is intracranial hypertension resulting from cerebral hemorrhage, leading to altered consciousness and systemic stress responses. This stimulates various humoral regulatory mechanisms, resulting in strong reactions. CRP and ALT can reflect the severity of the disease, and their elevation suggests a likely deterioration of the patient’s underlying condition, increasing the risk of worsening brain edema.

Based on these predictors, the GDBT model developed in this study demonstrated robust and consistent identification and calibration across the training, internal, and external validation cohorts. The selected results were interpretable and could be effectively applied in clinical practice. This model can potentially assist clinicians in identifying high-risk patients and informing clinical decision-making.

## Conclusion

The GDBT model consistently demonstrated superior performance in predicting 72-h changes in cerebral edema across the training, internal, and external validation cohorts. The SHAP and LIME analysis revealed that the first three favorable factors associated with increased edema (100%) included the following: sex (male), cerebral subarachnoid hemorrhage volume within the range of 0.02–0.15 mL, and a history of alcohol use. Conversely, the first three favorable factors associated with no increase in edema (99.8%) included the following: serum sodium levels exceeding 143.45 mmol/L, left parietal bleeding volume within the 0.13–0.67 mL range, and alanine aminotransferase levels between 19.04 U/L and 27.92 U/L. These findings have the potential to assist clinicians in the early identification of patients at risk for severe cerebral edema, enabling the implementation of targeted preventive measures to reduce its prevalence.

### Strengths

Our study has several advantages, including the inclusion of variables that closely reflect real-world human physiological conditions. The value of the volume of cerebral hemorrhage is collected until the hematoma disappears, no new hematoma occurs during the follow-up period, and the modeling process is a rigorous model development and a validation process, using multiple machine learning algorithms to identify the model with the best performance. Various evaluation measures and model interpretability techniques, such as SHAP and LIME, are used to ensure transparency and facilitate the interpretation of the results, and the model results can be effectively interpreted.

### Limitations

Despite its strengths, our study has several limitations. First, the sample size was not validated across multiple centers, and the ROC curve AUC of the constructed model was only 66%, potentially due to the inclusion of many complex and variable factors. The retrospective design of the study and the absence of some data may lead to the exclusion of potentially relevant predictors, such as hypoperfusion due to hypotension, high intracranial pressure, and ischemia or hypoxia due to blood pressure management based on arterial stenosis.

## Data Availability

The raw data supporting the conclusions of this article will be made available by the authors, without undue reservation.
