# Supplementary material for: Predicting cerebral edema in patients with spontaneous intracerebral hemorrhage using machine learning
Source: Front Neurol. 2024 Oct 3;15:1419608. doi: 10.3389/fneur.2024.1419608 (PMC11484451; doi:10.3389/fneur.2024.1419608)
Supplement: Supplementary file 1 [file Presentation_1.pdf]

Basic characteristics: sex, age, time(first imaging examination), stroke, diabetes, atrial fibrillation, coronary heart disease, hypertension, smoking, drinking, systolic pressure, diastolic pressure, hyperlipemia, decompressive craniectomy, cerebral ventricular drainage, hemostatic treatment, decreasing intracranial pressure, antihypertensive therapy, sedation, analgesic therapy, antiemesis and antacid, trophic nerve, hypolipidemic therapy, hemoglobin, blood platelet, count partial thromboplastin time, thrombin time, prothrombin time, triglyceride, cholesterol, HDL, ALT, CK, LDH, CK-MB, BNP, Mg, CGM, serum calcium, serum sodium, serum kalium, serum albumin, CRP, GCS-admission score, GCS-discharge score, ADL-admission score, ADL-discharge score, MRS-admission score, 180-days-NISS score, 180-day-MRS score, 180-days-GOS score(All but the follow-up data were data on the day of admission).

Imaging characteristics: hematoma volume, CT value1(hematoma), hematoma length (mm), hematoma short warp (mm), edema zone volume, CT value2(edema), the midline shift, intraventricular hemorrhage volume, CT value3(intraventricular), cerebral subarachnoid hemorrhage volume, CT value4(cerebral subarachnoid), subdural hemorrhage volume, CT value5(subdural), total hemorrhage volume; bleeding part: Brainstem, Left cerebellum, Left basal ganglia, Left frontal lobe, Left temporal lobe, Left thalamus, Left parietal lobe,

Left occipital lobe, Right cerebellum, Right basal ganglia, Right frontal lobe, Right temporal lobe, Right thalamus, Right parietal lobe, Right occipital lobel. Hemorrhage characteristics: Black hole, Mixture, Satellite lesion, Swirl sign; Shape score, Density, Lateral ventricle compression ratio.

SVM-RFE:Gender, Age, the stroke history, diabetes history, the hypertension history, alcohol history, the hyperlipidemia history, ventricular drainage, hemostatic treatment, decompressive craniectomy, antihypertensive treatment, antiemetic and gastric care, hypolipidemia treatment, hemoglobin, the platelet count, HDL, cholesterol, alanine aminotransferase, serum magnesium, serum sodium, CRP, ADL admission, ADL discharge, cerebral subarachnoid hemorrhage volume, subdural hemorrhage volume, CT value1, left cerebellum hemorrhage volume, left basal ganglia hemorrhage volume, left frontal lobe hemorrhage volume, left parietal lobe hemorrhage volume, right occipital lobe hemorrhage volume, fisher.

LASSO regression algorithm: Gender, time from onset to first imaging examination, diabetes history, history of coronary heart disease, hypertension history, alcohol history, decompressive craniectomy, hematoma removal, ventricular drainage, hemostatic treatment, craniohypotensive treatment, antihypertensive treatment, antiemesis and antacid, triglyceride, HDL, cholesterol, alanine aminotransferase, serum

magnesium, serum calcium, serum sodium, CRP, ADL admission, cerebral subarachnoid blood loss, subdural hemorrhage volume, left cerebellum volume, left basal ganglia volume, left parietal lobe volume, right frontal lobe volume.
